# Empirical Study on the Impact of Urban and Rural Resident Basic Medical Insurance on Children's Educational Outcomes

**DOI:** 10.3389/fpubh.2021.764364

**Published:** 2021-09-28

**Authors:** Hua Chen, Gandan Xiao, Jianing Xing, Qing Zhou

**Affiliations:** ^1^School of Insurance, Central University of Finance and Economics, Beijing, China; ^2^School of Finance and Economics, Guangdong Polytechnic Normal University, Guangzhou, China

**Keywords:** basic medical insurance, educational outcome, channel effect, double selection lasso, propensity score matching

## Abstract

This paper employs data from the 2016 and 2018 China Family Panel Studies (CFPS) to study the impact of participation in Urban and Rural Resident Basic Medical Insurance (URRBMI) on children's educational outcomes by using the logit model, double selection Lasso model, and propensity score matching. It is found that participating in URRBMI has no significant effect on children's Chinese performance, but has a significant negative effect on children's mathematics performance. The negative effect is more obvious for children who participating in the New Cooperative Medical Scheme (NCMS). The paper also studies the channel effects of participation in URRBMI on children's educational outcomes trough two different ways. It is noticed that both channel effects are not significant, that is, participation in URRBMI neither improves children's health nor changes household education expenditures.

## Introduction

Basic medical insurance is an important health care policy to guarantee residents' health. Basic medical insurance system in China consists of Urban employee medical insurance and Urban and Rural Resident Basic Medical Insurance (URRBMI). Urban employee medical insurance was established in 1998 and has formed a relatively standardized medical insurance system. According to the relevant documents, all urban employers, including enterprises, government office, public institutions, social organizations, private non-enterprise units, and their employees, are required to participate in Urban employee medical insurance. It is paid by the employer and employees in accordance with the national regulations, and a medical insurance fund is established. After the insured incurs medical expenses due to illness, the medical insurance agency will give the insured a certain amount of financial compensation to avoid or reduce the economic risk caused by the illness and treatment. The development of URRBMI is relatively late, Chinese government launched the New Cooperative Medical Scheme (NCMS) and Urban Resident Basic Medical Insurance (URBMI) in 2003 and 2007, respectively. URBMI is mainly for urban minors and residents who do not participate in Urban employees medical insurance, while NCMS is mainly for people who have rural registered permanent residence in China. The financial support for both comes from individual contributions and government subsidies. Since its establishment, Chinese government has undertaken a series of policy measures to expand the basic medical insurance coverage and improve security level. The coverage of basic medical insurance expands rapidly. According to data released by the National Medical Insurance Administration of China in 2020, by the end of 2019, basic medical insurance covers more than 95% of the population and has basically achieved universal coverage in China. However, due to its short history compared with other developed countries, especially the Urban and Rural Resident Basic Medical Insurance, and the great differences in the time and pattern of implementing policies among provinces, URRBMI still has some defects, such as insufficient compensation and strict restrictions on drug catalogs, which make the URRBMI unable to perform its due function. To address these problems, Chinese government and national medical insurance administration continue to adjust the current medical insurance policies to make the system give a full play.

As for study on the effect of the medical insurance policies, scholars usually start from improving health performance and smoothing household consumption, and main subjects are adults and the elderly. For example, studies focusing on the elderly population in the USA show that the expansion of Medicare coverage for the elderly at 65 age has led to more utilization of medical care, better self-reported health status, and lower out-of-pocket expenses but has little effect on mortality (1–3). Lei and Lin and Wagstaff et al. find no evidence that the NCMS has reduced out-of-pocket expenditure (4, 5). Cheng et al. use panel data from the Chinese Longitudinal Healthy Longevity Survey to evaluate the effects of the NCMS on health outcomes and spending of the rural elderly, results show that NCMS has significantly improved the ability of daily living and cognitive function of the elderly in rural areas, but it has no significant effect on the mortality rate of the elderly within 3 years (6). Some researches on children find that health insurance not only improves their health performance, but also has an impact on children's educational outcomes for the young enrollees. Studies have been carried out to evaluate the impact of health insurance on children's educational outcomes around the world. Levine and Schanzenbach use the difference-in-differences-in-differences strategy to evaluate the impact of public health insurance expansions on children's educational outcomes, including Medicaid and SCHIP. The educational outcomes are measured by 4th and 8th grade reading and math test scores, available from the National Assessment of Educational Progress. Results show that test scores in reading, but not math, increased for those children affected at birth by increased health insurance eligibility (7). Alcaraz et al. study the effect of a large expansion of Mexican public health insurance on school enrollment rates and on children's academic performance, using panel data from 2007 to 2010. They find that the expansion of public health insurance program has a large positive and statistically significant effect on school enrollment rates and standardized test scores (8). Chen and Jin find that the NCMS improves the school enrollment of children, however, since studied area is much poorer than most areas of China, the conclusion may not hold at the national level (9). Cohodes et al. show that expanding health insurance coverage for low-income children increases the rate of high school and college completion (10).

Study on the impact of basic medical insurance on children's educational outcomes has generally concluded that basic medical insurance affects children's educational outcomes through two main channels. Improving children's health is one direct channel. Scholars from home and abroad have examined health performances of health insurance. A number of studies show that the expansion of health insurance coverage has reduced child mortality (11–17). Wagstaff and Pradhan point out that Vietnam's national health insurance can improve the height and weight of school-age children (18). Bagnoli finds that the significantly positive impact of Ghana's National Health Insurance Scheme is concentrated among the lower-income households in regions with a high quality of public health care (19). Glewwe and Miguel and Currie examine how children's health status affect educational outcomes in developing and developed countries, respectively, results show that a increase in children's health level is associated with better future educational outcomes in both developed and developing countries (20, 21). Another channel is to reduce household uncertain expenditures, and thus families will increase their investment in children's education. Miller et al. find that health insurance can relieve the financial burden on households (22). Babiarz et al. find that enrollment in NCMS is associated with a decrease in out-of-pocket medical spending with the progress of the NCMS program (23, 24). Sun et al. measure the impact of NCMS on catastrophic medical payments of rural households in Linyi County, Shandong Province, and find that out-of-pocket health payments remain a severe burden for rural households (25, 26). Yip and Hsiao point out that expensive outpatient services for chronic conditions is the major reason why NCMS does not address medical impoverishment (27). Yip et al. find that financial protection provided by the NCMS is still limited (28).

Similar studies focusing on the impact of URRBMI on children's educational outcomes in China are limited. And the datasets they used in existing literatures are limited which can not reflect the real situation of the current stage of URRBMI. Our study will fill the gap of current research and enrich the research system for basic medical insurance system in China. In addition, improving the education level of children is one of the important ways to promote the country through science and education. But in recent years, because of the uneven economic development, the stratification trend of education resources in different classes and registrations become more obvious, and urban children have more access to quality education resources than rural children in their daily study. URRBMI has a redistributive effect on social wealth, and for rural children, if they can promote their own development by participating in URRBMI, it will be beneficial to the society. Therefore, while evaluating the role of the URRBMI system from multiple perspectives, this paper can provide implications for how to better improve the effect of the basic medical insurance system on children in the next reform.

This paper investigates the effects of URRBMI on children's educational outcomes in China, using data from the 2016 and 2018 waves of the China Family Panel Studies. It employs a strategy that combines propensity score matching with double selection lasso approach to address selection bias and endogenous problems. Results show that the URRBMI has significantly reduced the children's mathematics performance but has no significant effect on children's Chinese performance. It also finds that the negative effect is more obvious for children who participating in NCMS. Furthermore, it also studies the channel effects of how participating in URRBMI influences children's educational outcomes and finds that URRBMI neither improves children's health nor changes household education expenditures.

The rest of the paper is organized as follows. Section Data and Descriptive Statistics describes the data and descriptive statistics. Section Empirical Framework describes the empirical strategy. Section Empirical Results presents the results of the empirical analyses. Section Conclusion concludes the paper with a brief discussion.

## Data and Descriptive Statistics

### Data and Variables

The data we used in this paper is from Chinese Family Panel Studies (CFPS). CFPS is a national and comprehensive social tracking survey implemented by the Institute of Social Science Survey (ISSS) of Peking University. CFPS aims to collect data at the individual, household, and community levels to reflect the changes in China's society, economy, population, education, and health. Since children's database in 2014 and before does not contain the types of children's participation in medical insurance, the data we used in this paper starts from 2016. The children's database contains information on children aged 0–16, and children's questionnaires are mainly answered by their parents. The content of the questionnaire involves the children's physical characteristics, health status, nursing care, education, and other aspects. In addition, some older children also participate in personal questionnaire and provide more personal information, such as mental health, satisfaction with school, whether to hold a school position and other aspects. Excluding the children who have not yet attended school and the children who miss the key variables, this paper finally contains 6,105 samples, of which the sample size from the 2016 survey is 3,210 and the sample size from the 2018 survey is 2,895, of which 1,476 children participated in two survey rounds.

The explained variable in this paper is children's educational outcomes, and there is no uniform measurement standard of children's educational outcomes at present. Most scholars choose the enrollment rate as the metric, and some scholars choose the academic performance as the metric. Due to the implementation of 9-year compulsory education in China, most children under the age of 16 are in primary and junior high school, which are in compulsory education stage. Educational attainment or enrolment rates do not reflect real educational outcomes. Therefore, this paper uses two questions of “How is the child's performance in Chinese” and “How is the child's performance in mathematics” in the children's database to reflect children's current educational outcomes. For these two questions, the questionnaire has four levels of answers: excellent, good, medium, and poor. In this paper, children who answer “excellent” or “good” are considered to have good educational outcomes, and the value is 1. Otherwise the educational outcomes are poor, and the value is 0.

The core explanatory variable in this paper is “whether to participate in URRBMI,” and the value of the variable depends on the answer to the question of “medical insurance type” in the children's database. The value equals one if the individual participates in NCMS or URBMI. Otherwise, the value is 0. In addition, we construct two explanatory variables, “whether to participate in NCMS” for rural registered permanent residence children and “whether to participate in URBMI” for urban registered permanent residence children. The control variables in this paper include children's characteristics (e.g., health status, medical consumption, study status, etc.), parent-child relationships (e.g., whether to supervise children's study, expected education level of children, etc.), and household characteristics (e.g., household income, parents' age, education, etc.).

### Descriptive Statistics

#### Explained Variable

The descriptive statistics in Table 1 show that 5,421 of the 6,105 children have URRBMI and 684 have no URRBMI, accounting for only about 10% of the total sample size. In terms of means, there is no significant difference between the Chinese scores of insured children and uninsured children, while the mathematics scores of insured children are significantly lower than uninsured children at the 10% significance level, but if the significance level is set to 5%, it can be considered that the difference is not obvious.

**Table 1:** Descriptive statistics of the explained variable.

**Explained variable**	**Insured**		**Not insured**		**Differences**
	***N***	**Mean**	***N***	**Mean**	
Chinese	5,421	0.5728	684	0.5570	0.1576
Math	5,421	0.5794	684	0.6184	−0.039*

*Indicate that the coefficients significantly differ from 0 at the 10% levels.

#### Control Variables

The descriptive statistics of covariates in Table 2 include children's characteristics, parent-child relationships, and household's characteristics. Since CFPS database involves many aspects such as children and their household characteristics, and the number of variables that can be used in the empirical model exceeds 50, Table 2 only presents the descriptive statistics of some main control variables, and the standard deviation of the variable is shown in parentheses. In terms of children's characteristics, there is no significant difference in age and gender between insured and uninsured children, but more children with rural household registration account for insured children. In terms of physical condition, there is no significant difference in HAZ between insured and uninsured children, but the average WAZ of insured children is significantly lower than that of uninsured children. Children with medical insurance are more likely to seek active medical care when they are sick, and the children without medical insurance have a higher proportion of buying commercial health insurance, but there is no significant difference in total medical spending. In terms of average education spending, uninsured children spent more in the past 12 months. In terms of parent-child relationships, no matter whether the child participates in URRBMI or not, there is no significant difference in the expected education level of parents, but the parents of children without medical insurance will save more money for their children's future education and have a higher degree of academic supervision. In terms of household's characteristics, it can be seen that uninsured children have higher household income and their parents have higher educational level.

**Table 2:** Description of covariates and descriptive statistics.

	**Insured**	**Not insured**	**Differences**
**Children's characteristics**			
Gender:1 if male; 0 if female	0.5425 (0.5)	0.5409 (0.5)	0.0016
Age	10.8301 (2.57)	10.9781 (2.51)	−0.1480
Hukou: 1 if rural hukou; 0 if urban hukou	0.8624 (0.35)	0.6301 (0.51)	0.2323***
HAZ: Weight for Height Z-score	−0.2136 (0.79)	−0.2114 (0.79)	−0.0023
WAZ: Weight for Age Z-score	−0.0920 (0.84)	0.0233 (0.92)	−0.1153***
How to deal with illness: 1 if immediate medical attention; 0 others	0.6082 (0.49)	0.4942 (0.5)	0.1140***
Whether to purchase commercial medical insurance: 1 if purchase; 0 if not	0.1232 (0.33)	0.1681 (0.37)	−0.0449***
Total medical expenses	827.1188 (4750.1)	697.2515 (1784.03)	129.8673
Total education spending in the past year	3015.5970 (5775.85)	4403.5790 (8830.92)	−1387.98***
**Parent-child relationship**			
Desired level of education for children: 0 if no requirement; 1–8 if Primary to PhD	6.0212 (1.37)	5.9971 (1.36)	0.0241
Whether to save money for child's education: 1 if yes; 0 if no	0.1492 (0.36)	0.1813 (0.39)	−0.0321***
Whether the child is required to complete homework: “very often” to “never,” values 1–5	2.9439 (1.41)	2.8319 (1.42)	0.1121*
Whether to check your child's homework: “very often” to “never,” values 1–5	3.0624 (1.38)	2.9225 (1.4)	0.1398**
Whether to organize children to watch TV: “very often” to “never,” values 1–5	3.0042 (1.24)	2.9167 (1.23)	0.0876*
**Household's characteristics**			
Household income	50189.26 (180836.7)	62991.71 (237990.8)	−12802.45*
Father's age	36.5802 (16.02)	37.8626 (15.39)	−1.2824**
Father's education: “illiterate/semi-literate” to “PhD,” values 1–8	2.4950 (1.16)	2.7398 (1.37)	−0.2448***
Father's health status: “deceased” to “very healthy,” values 0–4	1.8510 (1.01)	1.9050 (1.04)	−0.0540
Mother's age	36.0057 (14.69)	35.6199 (15.51)	0.3858
Mother's education: “illiterate/semi-literate” to “PhD,” values 1–8	2.3156 (1.16)	2.5117 (1.36)	−0.1961***
Mother's health status: “deceased” to “very healthy,” values 0–4	1.8722 (1.06)	1.7909 (1.09)	0.0812*

*, **, and *** indicate that the coefficients significantly differ from 0 at the 10, 5, and 1% levels.

## Empirical Framework

### Logit Regression Model

This article mainly estimates the effect of the URRBMI on children's educational outcomes. Due to the explained variable to measure children's educational outcomes is a binary variable, the disturbance term is usually related to the explanatory variable when the explained variable is a discrete variable. In this situation, OLS is not suitable for estimation, so we employ the logit model for estimation:


(1) $$\begin{array}{l} {Perf}_{i}\;=\;\beta_{0}\;+\;\beta_{1}{Social\text{\_}ins}_{i}\;+\;\beta_{2}Z_{i}\;+\;\varepsilon_{i} \end{array}$$

Where *Perf*_*i*_ represents the performance of child *i* in Chinese or mathematics, *Perf*_*i*_ equals one if the child *i* in good educational outcomes and 0 otherwise. *Social*_*ins*_*i*_ is an indicator of whether the child *i* has URRBMI, *Social*_*ins*_*i*_ equals one if the child *i* has URRBMI and 0 otherwise. *Z*_*i*_ is a vector of covariates. Furthermore, in order to study whether there are differences in educational outcomes between children participating in NCMS and URBMI, we expand model (2) and model (3) on the basis of model (1), and explore the impact of NCMS and URBMI on children's educational outcomes, respectively:


(2) $$\begin{array}{lll} {Perf}_{i}\; & = & \;\alpha_{0}\;+\;\alpha_{1}{Xinnonghe}_{i}\;+\;\alpha_{2}Z_{i}\;+\;\tau_{i} \end{array}$$

(3) $$\begin{array}{lll} {Perf}_{i}\; & = & \;\eta_{0}\;+\;\eta_{1}{Chengjubao}_{i}\;+\;\eta_{2}Z_{i}\;+\;u_{i} \end{array}$$

### Double Selection Lasso Model

The data we use in this paper is high-dimensional data. *Z*_*i*_ is a vector of child's and household's covariates, in particular, the number of explanatory variables in the model will surge if we add quadratic term and cross multiplication term to the model. Putting all control variables into the model will lose degrees of freedom, and in practical research, we usually want to get a “sparse model,” that is, only the variables that have significant explanatory power to the model are selected, and the control variables with weak influence are ignored. Previous studies usually artificially selected control variables through references, data availability, and the purpose of this study. However, artificial selection is usually subjective, and important explanatory variables may be omitted. We use lasso regression to screen out the variables with strong interpretation of the model, and further logit estimation. The principle of lasso estimation is consistent with OLS regression, that is, minimizing the mean square error of estimation results. The difference is that lasso regression adds a penalty term to the OLS objective function to punish variables with large coefficient and further filter variables. The objective function of lasso regression is:


(4) $$\begin{array}{l} \hat{\beta }\;=\;{argmin}_{\beta }\left\{\sum_{i\;=\;1}^{N}{\left(Y-X\beta \right)}^{2}\;+\;\lambda \sum_{j\;=\;1}^{N}|\beta_{j}|\right\} \end{array}$$

Equation (4) consists of two terms. The first term is the objective function and the second term is the penalty term. λ indicates the penalty intensity, the higher λ represent the stronger penalty intensity, and the fewer variables to be screened. Machine learning usually uses cross validation to select λ. Due to lasso regression penalizes variables with large regression coefficients, the variables should be standardized to eliminate the errors caused by different dimensions of variables before lasso regression. The variables screened by lasso regression are usually used for data prediction rather than direct causal inference. One direct method is to bring variables directly into the logit model for coefficient estimation and causality inference. Chernozhukov et al. point out that separating variable screening and regression analysis will get wrong inference results, especially when the coefficient of an important explanatory variable is small, using this method will omit variables (29). In addition, Lasso regression is to screen variables according to the characteristics of the data itself, without considering the actual meaning of variables. It is also possible to omit variables, resulting in endogenous problems in model estimation. In this paper, we use the double selection lasso model proposed by Belloni et al. for logit estimation (30, 31).

The fundamental principle of double selection lasso model is perform two lasso regression on variables. First, lasso regression is performed on all control variables *Z*_*i*_ with the explained variable, and the selected control variables are recorded as $Z_{i}^{{\;}^{\prime }}$; Second, the core explanatory variable is used for lasso regression of all control variables, and the selected control variables are recorded as $Z_{i}^{{\;}^{\prime \prime }}$, the final logit estimation model is:


(5) $$\begin{array}{l} Perf_{i}=\;\beta_{0}\;+\;\beta_{1}Social\_ins_{i}\;+\;\beta_{2}Z_{i}{\;}^{\prime }+\;\beta_{3}Z_{i}{\;}^{\prime \text{​}\prime }\;+\;\varepsilon_{i} \end{array}$$

(6) $$\begin{array}{l} Perf_{i}=\;\alpha_{0}\;+\;\alpha_{1}Xinnonghe_{i}\;+\;\alpha_{2}Z_{i}{\;}^{\prime }+\;\alpha_{3}Z_{i}{\;}^{\prime \text{​}\prime }\;+\;\tau_{i} \end{array}$$

(7) $$\begin{array}{l} Perf_{i}=\;\eta_{0}\;+\;\eta_{1}Chengjubao_{i}\;+\;\eta_{2}Z_{i}{\;}^{\prime }\;+\;\eta_{3}Z_{i}{\;}^{\prime \text{​}\prime }+\;u_{i} \end{array}$$

After double lasso selection, important explanatory variables have been selected, and the possible endogenous problems of the model have been controlled.

### Propensity Score Matching

Due to take-up of the URRBMI is voluntary, we need to deal with the selection bias due to unobserved heterogeneity between enrollees and non-enrollees. Our main empirical strategy is using propensity score matching (PSM) approach proposed by Rosenbaum and Rubin for estimation (32). PSM matches enrollees and non-enrollees according to the covariates to construct the counterfactual of enrollees and remove selection bias. According to the potential results framework, *T*_*i*_ = 1 indicates that the individual has participated in the URRBMI, and *T*_*i*_ = 0 indicates that the individual has not participated in the URRBMI. *y*_1*i*_ refers to the potential results when participating in the URRBMI, and *y*_0*i*_ refers to the potential results when not participating in the URRBMI. In this case, if we assume that an individual satisfies the Conditional Independence Assumption (CIA), that is, the allocation of individual states and potential results are independent of each other, then *y*_1*i*_, *y*_0*i*_⊥*T*_*i*_|*X*_*i*_, *E*[*y*_0*i*_|*T*_*i*_ = 1] = *E*[*y*_0*i*_|*T*_*i*_ = 0], *E*[*y*_1*i*_|*T*_*i*_ = 1] = *E*[*y*_1*i*_|*T*_*i*_ = 0], the average treatment effect of enrollees would be estimated as:


(8) $$\begin{array}{l} \alpha_{ATT}=\;E\left[y_{1i}|T_{i}\;=\;1\right]-E\left[y_{0i}|T_{i}\;=\;1\right]\;=\;E\left[y_{1i}|T_{i}\;=\;1\right]\\ \;-E\left[y_{0i}|T_{i}\;=\;0\right] \end{array}$$

There are two prerequisites for use of PSM: (1) the conditional independence assumption and (2) the common interval assumption. Let *P*(*x*) = *Prob*(*T* = 1|*x*) be the propensity index, the common interval requires 0 < *Prob*(*T* = 1|*x*) < 1. Under these two assumptions, Rosenbaum and Rubin proved that *y*_1_, *y*_0_⊥*T*|*P*(*x*) ⊥ 0 < *Prob*(*T* = 1|*p*(*x*)) < 1. In this paper, we use the following equation to estimate the ATT based on matched samples.


(9) $$\begin{array}{l} \alpha_{ATT}\;=\;E_{p|T\;=\;1}\{E\left[y_{1}|T\;=\;1,p\left(x\right)\;=\;p\right]\\ \;-E\left[y_{0}|T\;=\;0,p\left(x\right)\;=\;p\right]\} \end{array}$$

## Empirical Results

### The Impact of URRBMI on Children's Educational Outcomes

#### Logit Regression and Double Selection Lasso Model

Logit regression and double selection lasso model are finally estimated by logit model. The difference is that the control variables are not screened in logit model, and the control variables are selected subjectively. In the double selection lasso model, the computer screens the variables according to the data characteristics. The model estimation results are shown in Table 3, and the values of the t-statistics are shown in parentheses.

**Table 3:** Logit and lasso regression.

	**Logit**		**LASSO**	
	**(1)**	**(2)**	**(3)**	**(4)**
	**Perf Chinese**	**Perf Math**	**Perf Chinese**	**Perf Math**
URRBMI	0.146 (1.61)	−0.176* (−1.87)	0.145 (1.61)	−0.169* (−1.82)
NCMS	0.140 (1.28)	−0.213* (−1.87)	0.153 (1.39)	−0.222** (−1.97)
URBMI	0.240 (1.29)	−0.127 (−0.66)	0.256 (1.45)	−0.067 (−0.37)

*, ** indicate that the coefficients significantly differ from 0 at the 10%, 5% levels.

Models (1) and (2) represent the estimation results of logit regression model. On the whole, having URRBMI has a positive trend to promote children's performance in Chinese, but the effect is not significant. By logit regression on the individuals participating in NCMS and URBMI, respectively. We can see that whether participating in NCMS or URBMI has a positive trend to promote children's Chinese performance, but the impact is not significant. However, it can be seen from model (2) that participating in URRBMI has a significant negative impact on children's mathematics performance, especially for children participating in NCMS, participating in URRBMI will make their mathematical performance worse than other rural registered permanent residence children who do not participate in NCMS. Models (2) and (3) represent the logit model estimation results after screening the control variables with double selection lasso model. The number of potential control variables reaches 5,982 after adding the square term and cross-multiplication term of control variables. In practice, the number of control variables obtained by each model after screening does not exceed 50. It can be seen from the results of models (3) and (4) that the results obtained by screening variables with lasso are similar to those of models (1) and (2), and the negative impact of participating in NCMS on children's mathematics performance is more significant. It can be considered that the results obtained by logit estimation after screening the control variables with double selection lasso model are reasonable and credible. When using high-dimensional survey data for research in the future, double selection lasso model can be used to screen control variables in order to prevent bias caused by artificial selection of variables, especially important cross-multiplication variables may be omitted.

#### Propensity Score Matching

To prevent endogeneity caused by self-selection, we further estimate the effect of URRBMI on children's educational outcomes after sample matching with PSM. We select the covariates for propensity score matching based on the method proposed by Imbens and Rubin (33), and then perform propensity score matching using the selected variables. There are various matching methods, and it is generally considered that the optimal matching method can minimize the variance after matching. After comparison, it is found that the matching method with a radius of 0.01 is the best and the variance reduced most after matching (34, 35).

Table 4 shows the estimation results of propensity score matching, and the values of the t-statistics are shown in parentheses. The first two columns of Table 4 show that participation in URRBMI has a significant positive effect on children's Chinese performance, but has no significant impact on mathematics performance. Columns 2 and 3 of Table 4 show the effect of whether to participate in NCMS on children's performance in Chinese and mathematics. It can be seen that participating in NCMS has no significant impact on children's Chinese performance, but has a significant negative impact on children's mathematics performance, while whether to participate in URBMI has no significant impact on children's Chinese and mathematics performance. The results are consistent with the conclusions obtained in Table 3 by using logit regression and double selection lasso model. The balance of the covariates is tested, and the results show that the covariates satisfy the balance assumptions.

**Table 4:** PSM regression.

	**URRBMI**		**NCMS**		**URBMI**	
	**Chinese**	**Math**	**Chinese**	**Math**	**Chinese**	**Math**
ATT	0.042* (1.81)	−0.024 (−1.13)	0.040 (1.48)	−0.046* (−1.75)	0.060 (1.45)	0.009 (0.23)
Average bias	1.6	1.2	1.4	1.5	2.8	3.5
Rubin's B	13.4	12.6	11.8	15.9	27	27.9
*N*	6,062	6,062	5,233	5,233	773	841

* indicates that the coefficients significantly differ from 0 at the 10% level.

### Robustness Analysis

#### Using Alternative Estimation Methods

In order to test the robustness of the results obtained by logit regression model, we use robust standard deviation and cluster standard deviation to estimate the logit models, and the results are shown in columns (1)–(4) of Table 5. Since the measurement indicators of children's Chinese and mathematics performance have ranking attributes, we also use ordinal logit regression model for robustness test. The results are shown in columns 5 (5) and (6) of Table 5. It can be seen that there is no significant difference between the results in Tables 3, 5. That is, participation in URRBMI has a potential positive effect on children's Chinese performance, but it has a significant negative effect on children's mathematics performance, especially participation in NCMS.

**Table 5:** Robustness test of logit model.

	**Robust standard deviation**		**Clustering standard deviation**		**ordinal Logit**	
	**(1)**	**(2)**	**(3)**	**(4)**	**(5)**	**(6)**
	**Perf Chinese**	**Perf Math**	**Perf Chinese**	**Perf Math**	**Perf Chinese**	**Perf Math**
URRBMI	0.145 (1.56)	−0.178* (−1.85)	0.145** (2.02)	−0.178** (−2.39)	0.146 (1.61)	−0.176* (−1.87)
NCMS	0.140 (1.23)	−0.214* (−1.81)	0.140 (1.48)	−0.214** (−2.07)	0.140 (1.28)	−0.213* (−1.87)
URBMI	0.245 (1.29)	−0.128 (−0.66)	0.245* (1.65)	−0.128 (−0.77)	0.240 (1.29)	−0.127 (−0.66)

*, ** indicate that the coefficients significantly differ from 0 at the 10%, 5% levels.

Secondly, in order to test the robustness of the results obtained by radius matching in the propensity score matching model, we use the one-to-one matching, one-to-four matching, and the one-to-four radius matching method to test the robustness. The matching results are shown in Table 6. Panel 1 to panel 3 show that participating in the NCMS has a significant positive effect on the children's Chinese performance, but it will weaken the children's mathematics performance, which is not significantly different from the results obtained in Table 4. Therefore, it can be considered that the results obtained by radius matching are robust.

**Table 6:** Robustness test of PSM matching methods.

	**URRBMI**		**NCMS**		**URBMI**	
	**Chinese**	**Math**	**Chinese**	**Math**	**Chinese**	**Math**
**Matching (1:1)**						
ATT	0.015 (0.52)	−0.008 (−0.3)	0.057* (1.70)	−0.049 (−1.54)	0.083* (1.73)	0.010 (0.21)
Average bias	2.1	2.7	2.7	2.7	4.6	5.7
Rubin's B	19.2	22.7	22.0	24.3	39.0	42.9
*N*	6,062	6,062	5,233	5,233	773	841
**Matching (1:4)**						
ATT	0.038 (1.50)	−0.018 (−0.80)	0.051* (1.75)	−0.052* (−1.87)	0.054 (1.28)	0.042 (0.02)
Average bias	1.4	1.5	1.7	1.7	2.9	4.5
Rubin's B	14.0	13.2	13.6	17.9	26.4	30.9
*N*	6,062	6,062	5,233	5,233	773	841
**Matching (1:4)radius**						
ATT	0.038 (1.50)	−0.018 (−0.78)	0.051* (1.75)	−0.052* (−1.88)	0.053 (1.25)	−0.004 (−0.10)
Average bias	1.3	1,5	1.7	1.8	3.0	4.6
Rubin's B	13.5	15.3	15.4	19.0	29.0	31.4
*N*	6,062	6,062	5,233	5,233	773	841

* indicates that the coefficients significantly differ from 0 at the 10% level.

#### Estimation by Subgroup of Children's Schooling Stage

In order to test whether the impact of URRBMI on the educational outcomes of children in different school stages is consistent, we perform propensity score matching at different school stages. Since the children in this study are aged 6–16 years old, most of them are in primary school and middle school, and a small number of them are enrolled in high school or technical vocational schools. Therefore, we divide the sample into two groups: primary and middle school. The results are shown in Table 7. It can be seen from panel 4 that participation in URRBMI has no significant effect on children's Chinese and mathematics scores in primary school. The reason may be that children at this stage mainly receive some basic knowledge and the gap between children is not significant. However, the results in panel 5 show that for children entering middle school and beyond, participation in URRBMI still has no significant effect on their Chinese performance, but it has a negative effect on their mathematics performance, which is significant at the significance level of 5%. Further analysis shows that the negative impact is mainly due to the fact that the mathematics performance of children participating in NCMS is significantly lower than that of children who are not covered by NCMS at the significance level of 5%. There is no significant effect of participation in URBMI on the performance of children in middle school and above.

**Table 7:** PSM estimates for different subgroups.

	**URRBMI**		**NCMS**		**URBMI**	
	**Chinese**	**Math**	**Chinese**	**Math**	**Chinese**	**Math**
**Panel 4:primary school**						
ATT	0.040 (1.49)	−0.001 (−0.05)	0.038 (1.24)	−0.013 (−0.42)	0.536 (1.34)	0.009 (0.19)
Average bias	1.6	1.3	1.5	1.6	3.9	3.4
Rubin's B	12.9	12.0	13.2	18.0	31.6	32.0
*N*	4,464	4,464	3,929	3,929	496	543
**Panel 5:middle school**						
ATT	0.071 (1.42)	−0.088** (−2.06)	0.057 (0.97)	−0.146** (−2.55)	0.043 (0.53)	0.039 (0.51)
Average bias	4.4	2.3	4.0	3.4	4.6	4.3
Rubin's B	31.8	19.2	31.0	26.6	37.9	2.9
*N*	1,593	1,593	1,240	1,299	277	298

** indicates that the coefficients significantly differ from 0 at the 5% level.

### Channel Effect

In order to investigate the mechanism of URRBMI on children's educational outcomes, combined with the existing literature, we evaluate the effect of URRBMI on children's educational outcomes through two channels: children's health status and household educational investment. We use HAZ, WAZ, and parents' health evaluation as the measurement indicators of children's health. The results of propensity score matching are shown in Table 8. It can be seen that whether using objective indicators or subjective indicators, participation in URRBMI does not improve children's health status. The results in Table 8 show that the channel effect of participating in URRBMI on children's educational outcomes through improving health status is not significant, so participation in URRBMI has no significant impact on children's educational outcomes.

**Table 8:** PSM estimates of URRBMI on children's health.

	**URRBMI**			**NCMS**			**URBMI**		
	**HAZ**	**WAZ**	**Parental evaluation** **of health**	**HAZ**	**WAZ**	**Parental evaluation** **of health**	**HAZ**	**WAZ**	**Parental evaluation** **of health**
ATT	−0.029 (−0.6)	−0.060 (−1.18)	−0.148 (−0.78)	−0.044 (−0.78)	−0.042 (−0.74)	−0.243 (−1.33)	0.023 (0.26)	0.051 (0.51)	0.479 (1.03)
Average bias	2.1	2.1	2.7	1.4	1.4	3.4	3.6	3.6	4.0
Rubin's B	15.0	15.0	24.0	12.3	12.3	19.6	27.5	27.5	32.0
*N*	6,062	6,062	3,165	5,233	5,233	2,810	773	773	324

In addition to improving children's health status, URRBMI will also affect household consumption by reducing uncertain household expenditure, especially investment in children's education, and thus affect children's educational outcomes. We use the total expenditure on children's education as the outcome variable. Because education expenditure presents a right biased distribution, we use the logarithm of education expenditure. The results are shown in Table 9. It can be seen that participating in URRBMI still has no significant impact on children's education investment, indicating that the effect of URRBMI on changing household education investment by reducing household uncertain expenditure is not significant. Therefore, the final results present that participation in URRBMI does not have a significant effect on children's educational outcomes, and even for children in rural households, participation in NCMS has a negative effect on their performance in mathematics.

**Table 9:** PSM estimates of URRBMI on children's educational outcomes.

	**URRBMI**	**NCMS**	**URBMI**
ATT	−0.021	−0.026	0.067
	(−0.21)	(−0.23)	(0.38)
Average bias	2.1	1.3	2.7
Rubin's B	15.2	12.3	27.4
*N*	6062	5233	773

The study of the above two channel effects shows that children's participation in URRBMI has no significant effect on their health and educational investment which may be the reason why there is no significant positive effect. Especially for rural registered permanent residence children, participation in the NCMS has a negative effect on their mathematics performance. This may be because participating in NCMS does not change their health and educational consumption. Convenient medical treatment may enable them to engage in other activities rather than study.

## Conclusion

Using the data of CFPS from 2016 and 2018 waves, this paper studies the impact of participating in URRBMI on children's education outcomes by using logit regression, double selection lasso, and propensity score matching method. Using lasso to screen control variables can effectively prevent possible errors caused by subjective selection. The main conclusions of this paper are as follows:

First, participation in URRBMI has a positive impact on children's Chinese performance, but this impact is not significant, but has a significant negative effect on children's mathematics performance. The reason why it has no significant impact on children's Chinese performance may be that the judgment of Chinese performance depends more on subjective judgment, while the judgment standard of mathematics performance is relatively objective, so the result is more significant. Participating in NCMS has no significant impact on children's Chinese performance, but has a significant negative impact on their mathematics performance. Participation in URBMI has a positive impact on children's Chinese and mathematics performance, but this impact is not significant.

Second, it is found that participating in URRBMI has no significant impact on academic performance of children in primary school by propensity score matching method, but has a significant negative impact on mathematical performance of children in middle school, which may be because children in middle school may engage in activities other than learning due to the convenience of medical services.

Thirdly, in order to study the mechanism of URRBMI on children's education outcomes, this paper studies from two aspects: children's health status and household education expenditure. It is found that that participation in URRBMI has no significant impact on children's health status and household education investment, indicating that the channel effect of participation in URRBMI on children's education outcomes is not significant, which confirms the conclusion that participation in URRBMI has no positive effect on children's educational outcomes.

It can be seen that although China's basic medical insurance system has been continuously reformed and optimized in recent years, the protection of children is still insufficient. URRBMI has not significantly improved their health status and education expenditure. Especially for children participating in NCMS, participating in NCMS has neither improved their health status nor educational outcomes. This result may be caused by two reasons: on the one hand, participating in NCMS does not reduce the financial burden of rural families, which leads to the inability to increase their investment in children's education. On the other hand, it may be that participation in NCMS makes it more convenient for middle school children to see a doctor, so they are more engaged in agricultural or other family activities, resulting in less learning and lower performance.

This paper not only makes up for the blank of research in China, but also expands the method of screening control variables. The method of selecting variables through lasso used in this paper can effectively solve the problem of control variable selection. However, since the data in this paper are only 2 years, this paper fails to study the impact of participating in URRBMI on children's long-term education outcomes. With the increase of available data in the future, scholars can study this issue. Besides, scholars can also measure children's educational outcomes from multiple perspectives, not limited to Chinese and mathematics performance.

In addition, we note that most regions in China have not designed and implemented a basic medical insurance program specifically for children, and children can only obtain medical coverage by participating in URRBMI or purchasing commercial health insurance, which do not help to improve their health status and access to other resources such as education. Therefore, in the next reform of the basic medical system, more attention should be paid to the improvement of children's effectiveness. It means that the future medical insurance reform should focus on effectively improving the economic situation of families, especially for rural families. We should not only improve the reimbursement proportion and coverage level of medical insurance blindly, but also pay more attention on education and publicity for rural families about the importance of education.

## Data Availability Statement

Publicly available datasets were analyzed in this study. This data can be found here: http://isss.pku.edu.cn/cfps/download/login.

## Author Contributions

All authors listed have made a substantial, direct and intellectual contribution to the work, and approved it for publication.

## Funding

We gratefully acknowledge the financial support from the National Natural Science Foundation of China (Grant No. 71974221) and the Humanities and Social Science Research Foundation of the Ministry of Education of China (Grant No. 19YJA630008).

## Conflict of Interest

The authors declare that the research was conducted in the absence of any commercial or financial relationships that could be construed as a potential conflict of interest.

## Publisher's Note

All claims expressed in this article are solely those of the authors and do not necessarily represent those of their affiliated organizations, or those of the publisher, the editors and the reviewers. Any product that may be evaluated in this article, or claim that may be made by its manufacturer, is not guaranteed or endorsed by the publisher.
